# Sensorial Systems Applied to Intelligent Spaces

**DOI:** 10.3390/s120810707

**Published:** 2012-08-06

**Authors:** José Luis Lázaro-Galilea, Ignacio Bravo-Muñoz, Alfredo Gardel-Vicente

**Affiliations:** Department of Electronics, Polytechnic High School, University of Alcalá, Alcalá de Henares 28871, Madrid, Spain; E-Mails: ibravo@depeca.uah.es (I.B.-M.); alfredo.gardel@uah.es (A.G.-V.)

The definition of “Intelligent Space” (IS) was formerly proposed for an environmental system capable of offering humans informative and physical support. Currently an IS denotes a space containing human and artificial systems, the space itself being considered an intelligent system. Human and artificial systems become clients of the IS and simultaneously the artificial systems become agents of the IS.

Since the whole space is an intelligent system, it is able to monitor and provide services to many different clients with ease. For example, an IS uses electronic displays to provide information to humans, then robots are used to offer physical services to them as physical agents.

When a robot lacks the sensors required to navigate around an IS, the robot is considered as a client and the rest of information is provided to the robot by the IS. An IS assumes two roles in relation to a robot working inside it. One is the enhancement of abilities, the other the sharing of resources.

Generally an intelligent robot has its own sensors and it is designed to move autonomously (without outside help). However, even an intelligent robot has limitations on its ability. Even though it has refined sensors, certain problems cannot be overcome. An IS has a role as an extended sensor for the robot and enhances the ability of the robot to receive requests from distant locations.

Resource sharing is valid when more than one robot uses the resources of an IS. Also, robots can reduce or eliminate such on-board resources as positioning sensors, sensors for detecting target objects, devices for interaction with humans, *etc*. However, an IS does not aim to make sensors redundant or reduce robot autonomy; rather, it supports robots with inadequate resources by providing the resources they lack in order to behave as a normal robot, while it helps robots with good resources to behave as even enhanced robots.

According to the IS definition, an IS should include different kind of sensors (possibly networked), a central node which will be processing the received data in order to take decisions and several actuators. Moreover, the sensors implemented in the majority of current systems have a certain level of autonomy and intelligence.

Nowadays, interest in IS is related to the facilities improvement in building automation systems such as residential houses, hospitals, schools, *etc*. Different proposals based on previous works are being developed in several research centers. Thus, new research fields close to IS are emerging such as pervasive computing, context-aware (environment), smart environment, smart houses, *etc*.

There are many challenges in this area that need to be solved or improved. The special issue “Sensorial Systems Applied to Intelligent Spaces” of *Sensors* journal seeks to explore new proposals in order to contribute in aspects such as: strategies, architectures, techniques and methods used in IS.

For this purpose, while several tens of manuscripts have been received, only 12 original and high quality manuscripts were selected to be included in this special issue. Each manuscript was reviewed by several reviewers—prestigious researchers in the same topics as the articles—and underwent up to three rounds of peer-review.

## Summary of the Special Issue

In addition to the current research line to develop hybrid systems (different kind of sensor technologies and localization systems) are emerging in the IS development and mainly in the works oriented to LPS (Local Position Systems) for IS, several proposals to improve the detection and localization inside IS, overcoming the achieved accuracy, decreasing the errors just as new applications of IS.

In this line, we would like to remark those published works in this Special Issue that their scope is different than the traditional point of view to use sensors just for a way to collect basic information from the room (temperature, presence, illumination level, *etc.*). Thus, we can emphasize works which propose techniques and methods for differential positioning, techniques to enhance the channel models in which the signals are propagated to be used in detection and positioning, strategies and distributed architectures to set up sensor networks in an IS, assessment of different indicators oriented to choose the best sensorial system to be used, methods for detailed localization of codified beacons and sensors within IS, movement effects in the detection/reception of signals, development of underwater LPS, design of an array of sensorial systems self-codified, *etc*. The majority of the proposed techniques presented in this Special Issue, are focused to applications in IS although some of them show interesting proposals in the infrared (IR), radio-frequency (RF) and wireless sensor networks (WSN) fields.

### Relevant Contributions Related to Ultrasounds (US)

The works submitted to this Special Issue related to US use as main technology for setting up an IS, we can find notable works such as the following:

In [1] a time-of-flight detection technique in the frequency domain is described for an ultrasonic LPS based on encoded beacons. A method based on the Generalized Cross-Correlation (GCC) for obtaining the Time Difference of Arrival (TDOA) of two signals coming from different beacons reaching up the same receiver has been proposed. Beacons emit encoded signals by means of Kasami sequences and positioning is achieved by using hyperbolic trilateration, based on the TDOA between a reference beacon and the others.

The detection algorithm is based on the cross-spectral density between the received signal and the modulated sequences to be detected. The algorithm has been tested with simulated and real data comparing its performance with that obtained when a basic correlation is used.

The work developed in [2] shows a method to calibrate the US beacons' positions in a LPS using a mobile receiver. These coordinates and the distances (or differences of distances) measured between the beacons and the mobile node to be localized are inputs to most trilateration algorithms. As a first approximation, such coordinates are obtained by means of manual measurements (a time-consuming and non-flexible method). In this proposal the location of only three test points must be known *a priori*, while the position of the other test points can be unknown. Furthermore, the paper describes a procedure to estimate the optimal positions, or approximate areas in the coverage zone, where the test-points necessary to calibrate the ultrasonic LPS should be placed.

The work in [3] analyses the effect of the receiver movement on the detection by pulse compression of different families of codes characterizing the emissions of an Ultrasonic Local Positioning System. It presents a detailed study of the influence that the receiver velocity can have on the matched filtering of the signals emitted by a particular ultrasonic LPS. Families of four BPSK modulated Kasami, LS and CSS sequences with different lengths have been considered in this study. Conclusions are that Complementary Set of Sequences are not the best choice when dealing with a moving receiver, since the auto-correlation bound of this family has a high value over the entire range of velocities; Kasami sequences seem to be a good choice when matched filtering of ultrasonic signals is used with a moving emitter/receiver.

In [4], an underwater acoustic propagation model is presented. The proposed model in this work obtains the multipath structure by means of the ray tracing technique. Using this model, the behavior of a relative positioning system is presented. One of the main advantages of relative positioning systems is that only the distances between all the buoys are needed to obtain their positions. Multipath caused by low wind speeds has been identified as the most damaging effect, and some outliers have been detected, due to the near-far effect, which disguise the behavior of the system. As an early example of an application using this relative positioning system, a tracking of the position of the buoys at different times is performed.

In [5] authors present the design of an ultrasonic array for obstacle detection based on Phased Array (PA) techniques, which steers the acoustic beam through the environment by electronics rather than mechanical means. The transmission of every element in the array has been encoded (with a pseudo orthogonal Kasami codes for each direction), according to Code Division for Multiple Access (CDMA), which allows multiple beams to be transmitted simultaneously and achieves longer inspection distances in comparison with conventional PA techniques. Thanks to the encoded emission, the SNR significantly increases when compared to conventional PA algorithms.

### Relevant Contributions Related to RF and WSN

The research lines in which a fusion of RF and WSN is used are growing up during last years. Thus, the different proposals to apply and improve the efficient use of this kind of networks, has had a notorious evolution. In this Special Issue the published works which include newest and relevant topics such as: development of techniques for increasing the positioning robustness depending of the handicaps and problems related to the channels, proposals of network sensors (RF and Wi-Fi) based on fuzzy rules optimizing and improving the problems and inaccuracies of current localization techniques, using fuzzy rules to get the position.

The work in [6] proposes two weighted least squares techniques based on the standard hyperbolic and circular positioning algorithms that specifically consider the accuracies of the different measurements to obtain a better estimation of the position. Signal Strength localization techniques using propagation channel models are the simplest alternative, but they usually consider that the radio propagation model is perfectly characterized. This algorithms produce better localization results with a very limited overhead in terms of computational cost and achieve a greater robustness to inaccuracies in channel modeling (on different types of wireless networks: WSN, Wi-Fi and Bluetooth networks). In a real deployment the hyperbolic algorithms have the advantage of a lower computational cost; where the channel estimation may not be very accurate, the use of this positioning algorithm may definitively improve the localization system performance.

In [7] a distributed architecture proposal for collaborative Fuzzy Rule-Based Systems embedded in WSNs has been presented, which has been designed to optimize the implementation of ISs. In this proposal authors pay attention in several aspects of the architecture such as: an optimized design for the inference engine; a visual interface; a module to reduce the redundancy and complexity of the knowledge bases; a module to evaluate the accuracy of the new knowledge base; a module to adapt the format of the rules to the structure used by the inference engine and a communications protocol.

The results suggest that the architecture presented in this paper allows a decrease in the consumption of the resources required (memory, CPU and battery) by an FRBS embedded in a node of a WSN, without a substantial decrease in the accuracy of the inferred values.

Others contributions in the SI tray to solve the problem consisting of locating a mobile node using only inter-node range measurements estimated by radio frequency signal strength attenuation. In [8] authors present the development and experimental evaluation of a method based on fuzzy logic to locate mobile robots in an IS using WSNs. This approach is focused on simple setting and robustness.

The use of on-site startup by simply tuning the approximate sensor models supposes an important advantage with respect to popular WSN localization approaches based on signal strength calibration because the setting of these systems is complex and time consuming.

The authors compare this approach with a probabilistic technique showing that the fuzzy approach is able to handle highly uncertain situations that are difficult to manage by well-known localization methods.

### Relevant Contributions Related to IR, IMU and Generic Techniques

In addition to the research lines described above, there are outstanding works that use less explored alternatives or improved techniques that can be used regardless of the sensor technology chosen in the IS.

In [9] a sensorial system for measuring differential phase-shifts in a sinusoidal modulated infrared signal transmitted from the robot has been presented. Differential distances were obtained from these phase-shifts, and the position of the robot was estimated by hyperbolic trilateration. The strong trade-off between coverage, precision and real time performance of the system has been solved by carefully designing the receiving and signal treatment stages. This renders it possible to recover phase information from very low quality signals, providing an effective SNR improvement of up to 37.5 dB. In addition, I/Q demodulation allows strong filtering at the output, which drastically reduces noise. Improved precision could be achieved by stronger filtering but at the cost of slower response, so that real time performance would be worse.

In [10] it is shown a new purpose for people localization in indoor environments different one that the partial solutions based on the deployment of a network of sensor. This solution is based on Pedestrian Dead-Reckoning (PDR) and only requires the installation of an Inertial Measurement Unit (IMU), on the person's body. The main problem of PDR is the accumulation of positioning errors due to the drift caused by the noise in the sensors. The proposed solution incorporates a drift correction method based on detecting the access ramps usually found in buildings. After detection, the ramp is checked for association with one of the existing in a database. For each associated ramp, a position correction is fed into the Kalman Filter in order to refine the INS-PDR solution. Drift-free localization is achieved with positioning errors below 2 meters for 1,000-meter-long routes in a building with a few ramps.

The work presented in [11] proposes a method to estimate the propagation of the errors of the inter-beacon distances obtained with the Linearized Auto-Localization (LAL) algorithm, The method is based on the differential sensitivity analysis that uses a first order Taylor approximation to obtain the function's error variance; in this work a confidence parameter was defined to measure the reliability of the estimated error distribution.

The LAL algorithm estimates the position of beacon nodes in Local Positioning Systems (LPSs), using only the distance measurements to a mobile node whose position is also unknown and calculates the inter-beacon distances, used for the estimation of the beacons’ positions, from the linearized trilateration equations. The differential sensitivity analysis shows that the standard deviation of the solution, obtained by the LAL method, is proportional to the standard deviation of the distance measurements. This work develops two versions of the error estimation: the offline method and the online method.

Work developed in [12] outlines the idea of IS for the sports context with special focus to game sports and how intelligent sports feedback systems can benefit from IS. It could provide information, insights and perspectives for the day-to-day work of coaches and players and thereby ensure high unobtrusiveness and usability. This information has to meet the needs and intentions of the actors and thus should assist them in their practical work for measuring the amounts of physical activities as well as for tactical assessments in game sports.

This work reviews the most common location sensing techniques used in sports and their practical application, as location is among the most important enabling techniques for IS. Authors conclude that in the present framework conditions, vision is a good solution for providing proper raw data, despite of some important aspects that makes it inefficient.
